# Biobanking for glomerular diseases: a study design and protocol for KOrea Renal biobank NEtwoRk System TOward NExt-generation analysis (KORNERSTONE)

**DOI:** 10.1186/s12882-020-02016-z

**Published:** 2020-08-26

**Authors:** Eunjeong Kang, Yaerim Kim, Yong Chul Kim, Eunyoung Kim, Nankyoung Lee, Yeonghui Kim, Soojin Lee, Seungyeup Han, Misun Choe, Jin Ho Hwang, Sunhwa Lee, Ji In Park, Jung Tak Park, Beom Jin Lim, Jung Pyo Lee, Jung Nam An, Dong-Ryeol Ryu, Jung-Hyun Kim, Hee Gyung Kang, Hyun Soon Lee, Kyung Chul Moon, Kwon Wook Joo, Kook-Hwan Oh, Seung Seok Han, Hajeong Lee, Dong Ki Kim, on behalf of the KORNERSTONE Study Group, Jung Pyo Lee, Jung Nam An, Jeonghwan Lee, Jeonghwan Park, Minjung Kim, Taekyoung Kim, Jinhyuk Kim, Jin Ho Hwang, Eun A. Park, Eunji Park, Ji In Park, Sun Hwa Lee, Soyeong Park, Nayoung Koh, Seungyeup Han, Yaerim Kim, Misun Choe, Yeonghui Kim, Dong Ki Kim, Kwon Wook Joo, Kook-Hwan Oh, Hajeong Lee, Seung Seok Han, Yong Chul Kim, Eunjeong Kang, Soojin Lee, Kyung Chul Moon, Hee Gyung Kang, Eunyoung Kim, Junghee Kim, Ji Hye Park, Ji Won Jeon, Jung Tak Park, Beom Jin Lim, Hyung Woo Kim, Young Su Joo, Kyungjoon Kim, Bo Young Nam, Eunyoung Kim, Nankyoung Lee

**Affiliations:** 1 Department of Internal Medicine, Ewha Womans University Seoul Hospital, Ewha Womans University College of Medicine, Seoul, South Korea; 2 Department of Internal Medicine, Keimyung University School of Medicine, Daegu, South Korea; 3 Department of Internal Medicine, Seoul National University Hospital, Seoul National University College of Medicine, Seoul, South Korea; 4 Seoul National University Hospital Clinical Trial Centre, Seoul, South Korea; 5 Seoul National University Hospital Human Biobank, Seoul, South Korea; 6 Division of Nephrology, Department of Internal Medicine, Keimyung University Dongsan Hospital, Daegu, South Korea; 7 Department of Pathology, Keimyung University School of Medicine, Daegu, South Korea; 8 Department of Internal Medicine, Chung-Ang University Hospital, Seoul, South Korea; 9 Division of Nephrology, Department of Medicine, Kangwon National University Hospital, Kangwon National University School of Medicine, Chuncheon, Gangwon-do South Korea; 10 Department of Internal Medicine, College of Medicine, Institute of Kidney Disease Research, Yonsei University, Seoul, South Korea; 11 Department of Pathology, Yonsei University College of Medicine, Seoul, South Korea; 12 Department of Internal Medicine Seoul National University Boramae Medical Center, Seoul National University College of Medicine, Seoul, South Korea; 13 Department of Internal Medicine, Hallym University Sacred Heart Hospital, Anyang, South Korea; 14 Department of Home Economics Education, Major of Food and Nutrition, Pai Chai University, Daejeon, South Korea; 15 Department of Paediatrics, Seoul National University Children’s Hospital, Seoul National University College of Medicine, Seoul, South Korea; 16 Department of Pathology, Hankook Renal Pathology Lab, Seoul, South Korea; 17 Department of Pathology, Seoul National University Hospital, Seoul National University College of Medicine, Seoul, South Korea

**Keywords:** Nephrology, Pathology, Glomerulonephritis

## Abstract

**Backgrounds:**

Glomerular diseases, a set of debilitating and complex disease entities, are related to mortality and morbidity. To gain insight into pathophysiology and novel treatment targets of glomerular disease, various types of biospecimens linked to deep clinical phenotyping including clinical information, digital pathology, and well-defined outcomes are required. We provide the rationale and design of the KOrea Renal biobank NEtwoRk System TOward Next-generation analysis (KORNERSTONE).

**Methods:**

The KORNERSTONE, which has been initiated by Korea Centres for Disease Control and Prevention, is designed as a multi-centre, prospective cohort study and biobank for glomerular diseases. Clinical data, questionnaires will be collected at the time of kidney biopsy and subsequently every 1 year after kidney biopsy. All of the clinical data will be extracted from the electrical health record and automatically uploaded to the web-based database. High-quality digital pathologies are obtained and connected in the database. Various types of biospecimens are collected at baseline and during follow-up: serum, urine, buffy coat, stool, glomerular complementary DNA (cDNA), tubulointerstitial cDNA. All data and biospecimens are processed and stored in a standardised manner. The primary outcomes are mortality and end-stage renal disease. The secondary outcomes will be deterioration renal function, remission of proteinuria, cardiovascular events and quality of life.

**Discussion:**

Ethical approval has been obtained from the institutional review board of each participating centre and ethics oversight committee. The KORNERSTONE is designed to deliver pioneer insights into glomerular diseases. The study design allows comprehensive, integrated and high-quality data collection on baseline laboratory findings, clinical outcomes including administrative data and digital pathologic images. This may provide various biospecimens and information to many researchers, establish the rationale for future more individualised treatment strategies for glomerular diseases.

**Trial registration:**

NCT03929887.

## Background

The prevalences of chronic kidney disease (CKD) and end-stage renal disease (ESRD) are continuously increasing around the world. Recent evidence indicates that the number of incident ESRD patients due to glomerular diseases is increasing [1, 2], and many additional ESRD cases are expected to have been attributed to glomerular diseases, considering the cases that have not been confirmed by kidney biopsy. However, the discovery of pathognomonic biomarkers and disease-specific therapeutic targets for glomerular diseases is limited.

To better understand the pathophysiology, biomarkers, and treatment targets of glomerular diseases and ultimately to improve clinical outcomes, it is imperative to translate and integrate research with various types of biospecimen linked to deep clinical phenotyping, including clinical information, self-questionnaires, digital pathology and outcomes from the electronic health record (EHR) and administrative data. A significant additional number of kidney disease cohorts were established through several international consortiums. The C-PROBE, [3] ERCB cohort [4] with CKD patients, and Nephrotic Syndrome Study Network (NEPTUNE) cohort [5] for patients with nephrotic syndrome were started in approximately 2010, and these cohorts are relatively small. Recently, large-scale genomics consortia, including CKDGen [6] and EURenOmics, [7] have been activated in the United States and Europe. However, these cohorts are not sufficient to support data for Asians. To that end, a multicentre prospective cohort based on deep clinical phenotypes, biospecimens, and digital pathology has been established in Korea, the ‘KORNERSTONE’, which is supported by the Korea Centers for Disease Control and Prevention (KCDC).

The objectives of the KORNERSTONE are as follows: 1) establish a collaborative, innovative deep clinical phenotype-based investigational infrastructure to carry out clinical and translational studies of glomerular disease; 2) perform a longitudinal observational cohort study of patients with biopsy-proven glomerular diseases; and 3) gain insight into the pathophysiology, natural history and novel treatment targets of glomerular diseases. In the present paper, we report the KORNERSTONE design and methods in detail.

## Methods/design

### Ethics statements

The basic protocol of this study was approved by the ethics committee of each participating centre, including the institutional review boards of Seoul National University (1404–117-575), Keimyung University Dongsan Hospital (DSMC 2019–04–015-001), Chung-Ang University (1942–005-369), Severance Hospital (2019–0463-001), Boramae Medical Center (L-2019-126), and Kangwon National University Hospital (KUNH-2019-05-009). Additionally, the ethics oversight committee of the KORNERSTONE reviewed the design and protocols of this study. All of the ethical issues regarding the plans of the KORNERSTONE cohort study will be supervised by this committee.

### Study design and population

The KORNERSTONE is a prospective multicentre observational biobank, databank and digital pathology repository of patients with glomerular disease in Korea (NCT 03929887 at http://www.clinicaltrials.gov). Nephrologists working in six clinical centres in the major university-affiliated hospitals, pathologists, and paediatricians are participating in this study. Among the six clinical centres, four centres are located in the metropolitan city of Seoul, one is in the southern area, and the last centre is in northeastern Korea.

Patients suspected of glomerular diseases who received kidney biopsy in participating university medical centres are eligible for inclusion in the KORNERSTONE. Patients who previously received a kidney transplant will be excluded. This study will enrol 3000 children (age < 18 years) and adults. Eligible participants will be screened at the inpatient or outpatient clinic of the department of nephrology or paediatrics of each participating hospital. Participants who pass an initial screening and agree to participate will provide written informed consent during their visit. Parents of all children, and children themselves if age more than 6 years, provided written informed consent. Informed consent includes utilization, collection, and provision of demographics, clinical information, and social security number for the linkage of administrative data and biospecimens. When a participant wants to withdraw from the KORNERSTONE, all stored biospecimens and data will be destroyed or deleted. All of the laboratory data, self-questionnaires and biospecimens will be collected at the time of kidney biopsy for the baseline information (Table 1). Patients or the public were not involved in the design, or conduct, or reporting, or dissemination plans of our research.

**Table 1:** List of clinical information and biospecimens in the KORNERSTONE

Parameters	Detailed information	Screening	Baseline	1 Y	2 Y	3 Y	4 Y	5 Y	Improvement or deterioration of clinical outcomes	Hard outcomes (ESRD, CV outcomes)
**Informed consent**		O								
**Demographic information**		O								
**Medical history**		O								
**Medications**	Hypertensive medications		O	O	O	O	O	O	O	O
	Immunosuppressant		O	O	O	O	O	O	O	O
**Questionnaires**	Health questionnaires (including smoking)		O	O	O	O	O	O	O	O
	KDQOL-SF version 1.3 questionnaires		O	O	O	O	O	O	O	O
	Semi-food frequency questionnaires		O	O	O	O	O	O	O	O
	PedsQL 4.0 Generic Core Scales (Children)		O							O
**Anthropometry**	Height, weight		O	O	O	O	O	O		O
	Blood pressure		O	O	O	O	O	O		O
**Blood laboratory findings**	CBC (white blood cell count, platelet, haemoglobin)		O	O	O	O	O	O	O	O
	Chemistry (calcium, phosphorus, glucose, blood urea nitrogen, uric acid, total protein, albumin, total bilirubin, alkaline phosphatase, AST, ALT)		O	O	O	O	O	O	O	O
	IDMS-traceable Cr, eGFR		O	O	O	O	O	O	O	O
	Lipid panel (total cholesterol, triglyceride, high-density lipoprotein, low density lipoprotein)		O	O	O	O	O	O	O	O
	Electrolyte panel (sodium, potassium, chloride)		O	O	O	O	O	O	O	O
	Total CO_2_		O	O	O	O	O	O	O	O
	C-reactive protein		O	O	O	O	O	O	O	O
	Serology (HBsAg, HBsAb, Anti-HCV, HIV, VDRL)		O							
	Anti-dsDNA, FANA, FANA titre, ANCA, PR3, MPO		O							
	Complement 3, 4		O							
	ASO, rheumatoid factor, Cryoglobulin		O							
**Urine laboratory findings**	Random urine protein		O	O	O	O	O	O	O	O
	Random urine microalbumin		O	O	O	O	O	O	O	O
	Random urine creatinine		O	O	O	O	O	O	O	O
	Urinalysis protein		O	O	O	O	O	O	O	O
	Urinalysis RBC		O	O	O	O	O	O	O	O
**Kidney biopsy**	Biopsy results and images including LM, IF and EM		O							
	Report of diagnosis		O							
	Total number of glomeruli		O							
	Total number of global sclerosis/segmental sclerosis/crescent		O							
**Biospecimens**	Serum/plasma/buffy coat/genomic DNA sample		O	O	O	O	O	O	O	O
	Urine sample		O	O	O	O	O	O	O	O
	Stool sample		O							
	cDNA from glomeruli and tubules in micro-dissected kidney biopsy tissues		O							

*CBC* complete blood count; *KDQOL-SF* Kidney Disease-Quality of Life Short Form; *PedsQL* Pediatric Quality of Life Inventory; *AST* aspartate aminotransferase; *ALT* alanine aminotransferase; *HCV* hepatitis C virus; *HIV* human immunodeficiency virus; *VDRL* veneral disease research laboratory test; *FANA* fluorescent antinuclear antibody; *MPO* myeloperoxidase; *ASO* antistreptolysin O; *RBC* red blood cell; *LM* light microscopy; *IF* immunofluorescent; *EM* electron microscopy; *IDMS* isotope dilution mass spectrometry; *Cr* creatinine; *eGFR* estimated glomerular filtration rate; *Y* year

### Sample size consideration

Table 2 summarizes the statistical power for time-to-event analyses (minimum detectable hazard ratio [MDHR]). For illustration, MDHRs were described for comparisons between two groups of equal size, which could represent any subgroups of interest (e.g., genetic risk alleles, treatment exposures, and clinical diagnosis). Clinical outcome event rates, including ESRD, death and complete remission, were based on published articles and early observed data in other glomerular disease cohorts [8–11].

**Table 2:** Minimum detectable hazard ratio for time-to-event outcomes

Outcome	5-year event rates	MDHR^**a**^ for ***n*** = 3000 (1500/group)
ESRD	0.12	1.36
Death	0.06	1.54
Complete remission of proteinuria (< 0.3 g/day)^b^	0.63–0.97	1.14–1.17

*Abbreviations*: *MDHR* minimum detectable hazard ratio; *ESRD* end-stage renal disease

^a^MDHR is based on the following assumptions: patient follow-up time of 5 years (ESRD and death); a loss of follow-up rate during 5 years hypothesized total of 10%; 80% power, alpha = 0.05

^b^Group sizes for time to complete remission of proteinuria excluded one-third of the group that was in remission at enrolment

### Clinical, patient-oriented outcomes and follow-up

After enrolment, all of the participants will be assessed for whether they reach clinical endpoints each time they visit the outpatient clinic. The clinical endpoints of this study were divided into two categories: 1) deterioration of clinical outcomes was defined by doubling of serum Cr or decrease in estimated glomerular filtration rate (eGFR) by more than 30%, and 2) improvement of clinical outcomes was defined by the remission of glomerulonephritis and proteinuria < 0.3 g/day. Other hard outcomes were defined by ESRD, cardiovascular events, and death. The patients who have proteinuria < 0.3 g/day at baseline have no improvement in clinical outcomes.

Whether the enrolled patients reached each clinical endpoint will be confirmed by the EHR. If the participant displays deterioration, improvement of clinical outcomes, ESRD or cardiovascular events, laboratory findings, urinalysis, and biospecimens, including serum, plasma, buffy coat, and urine, will be collected from the participants. Stools will be collected at baseline only. Additionally, the same information and biospecimens will be collected from the participants at least once per year.

In addition, the patient-oriented outcome will be secured by age-appropriate questionnaires. These outcomes will include symptoms from the patient’s perspective, functional status, nutritional status and health-related quality of life. The questionnaires will be collected every year.

**Fig. 1:**
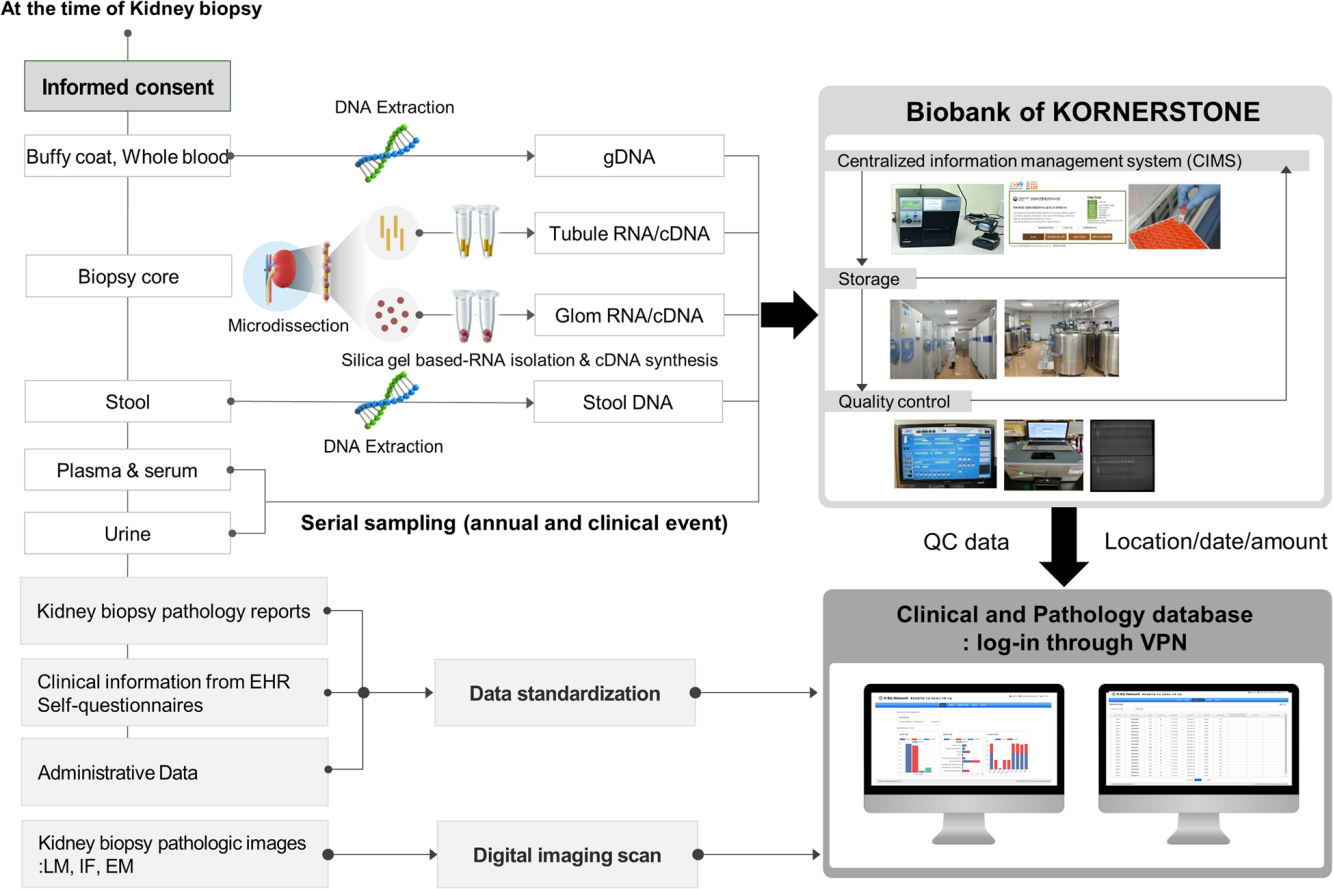
Overall schemes of the KORNERSTONE. KORNERSTONE, Korea Glomerular disease Biobank Network; gDNA, genomic DNA; OPD, outpatient; EHR, electronic health record; LM, light microscopy; IF, immunofluorescence; EM, electron microscopy; QC, quality control; VPN, virtual private network

All of the administrative data from Statistics Korea, Health Insurance Review and Assessment Service (HIRA) and National Health Insurance Service (NHIS) and ESRD registry data from the Korean Society of Nephrology will be linked through social security numbers or birth date. However, the information will not be stored by the researchers and will only be used for the collection of clinical outcome information under the approved protocol. The prescribed medication and diagnosis defined by the 10th revision of the International Statistical Classification of Diseases and Related Health Problems (ICD-10) will be collected by HIRA and NHIS. The mortality and the cause of death will be obtained from Statistics Korea data. The reached ESRD outcomes, including dialysis and kidney transplantation, will be determined using the data from NHIS and the Korean ESRD registry from the Korean Society of Nephrology (Fig. 1). For clinical hard outcomes, including death and ESRD, the EHR of each participating centre and various administrative data will be used, so the outcome can be reliably determined even in the case of patient transfer to another hospital or of patients who cannot participate this cohort for other reasons.

### Electronic health record and questionnaires

The detailed protocol for clinical data and questionnaire collection is described in Table 1. The eligible patients will be evaluated at baseline for demographic information, smoking history, chief complaints, and anthropometric information, including height, weight, and blood pressure. Measurements of the resting blood pressure in the inpatient setting on the kidney biopsy date using an electronic sphygmomanometer will be carried out.

We will collect laboratory findings closest to the kidney biopsy date. Serum creatinine will be measured using an isotope dilution mass spectrometry (IDMS) traceable method. The eGFR will be calculated using the Chronic Kidney Disease Epidemiology Collaboration (CKD-EPI) equation for adults. In children younger than 18 years, the creatinine-cystatin C-based CKiD (Chronic Kidney Disease in Children) equation will be used. Blood laboratory tests, such as complete blood cell count; chemistry, including calcium, phosphorus, total protein, and albumin; lipid panel; electrolyte panel; viral serology, and glomerular disease-related serology will be conducted. Quantitative tests of proteinuria and albuminuria will also be carried out. The information on prescribed medication will include the start and end date of each medication, doses per der day, and total number of prescription days.

The subjects will complete age-appropriate questionnaires concerning the quality of life using Kidney Disease and Quality of Life Short Form (KDQOL-SF) version 1.3, socioeconomic status, educational level, physical activity, health behaviours, health care facility utilization, and nutritional status using semi-food frequency questionnaires (semi-FFQs) and a food diary.

### Human biospecimen collection, quality control and standard operating procedure (SOP)

Eight types of biospecimens will be collected: plasma, serum, buffy coat, genomic DNA, urine, stools, stool DNA and cDNA from glomeruli and tubules in microdissected kidney biopsy tissues (Table 1). Anonymous barcodes will be printed and attached to all samples collected. Additionally, registered information, including the type, quantity, and location of biospecimens, will be uploaded to the web database. All of the samples will be subsequently collected regularly according to the standardized protocol. All of the biospecimens, including plasma, serum, buffy coat, urine, genomic DNA and stool DNA, will be stored at the National Biobank of Korea launched by the KCDC.

Blood will be stored as plasma, serum, buffy coat, and genomic DNA. From one participant in the KORNERSTONE, at least one SST tube and two EDTA tubes with blood (8 cc per tube, total 24 cc), more than 10 cc of urine, more than 1 g in 2 bottles of stool, stool DNA, and at least one core of kidney biopsy tissue will be collected. Blood and urine samples will be centrifuged for 10 min and will be distributed in cryotubes. Serum and plasma will be stored in a liquid nitrogen freezer (− 196 °C), and other types of biospecimens will be stored in a deep freezer (− 80 °C) (Table 3).

**Table 3:** Overview of stored samples per participant in the KORNERSTONE

Biospecimen	Stored vial	Volume and amount	Storage temperature
Serum	1.8 ml cryogenic vial	Adults: 300 μl × 5 vials	≤ − 196 °C
		Children: 100 μl × 4 vials	
Plasma	1.8 ml cryogenic vial	Adults: 300 μl × 5 vials	≤ − 196 °C
		Children: 100 μl × 4 vials	
Genomic DNA	1.7 ml microcentrifuge tube	Adults: Total more than 100 μg, 3 vials	≤ − 80 °C
		Children: Total 30 μg, 2 vials	
Urine	1.8 ml cryogenic vial	Adults: 1 ml × 5 vials	≤ − 80 °C
		Children: 200 μl × 5 vial	
Buffy coat	1.8 ml cryogenic vial	Adults: 200 μl × 2 vials	≤ − 80 °C
		Children: 100 μl × 2 vials	
Stool DNA	1.7 ml microcentrifuge tube	100 μl, 1 vial	≤ − 80 °C
Stool	30 ml container	1 g, 2 bottles	≤ − 80 °C
Microdissected glomerulus (cDNA)	1.7 ml microcentrifuge tube	Total 70 ng × 2 vials	≤ − 80 °C
Microdissected tubulointerstitium (cDNA)	1.7 ml microcentrifuge tube	Total 100 ng × 2 vials	≤ − 80 °C

For quality control of biospecimens, 7-digit Standard PREanalytical Code (SPREC), which encodes pre-processing information of biospecimens, will be assigned for each blood and urine sample [12]. Serum and plasma samples will be visually checked for haemolysis. The protocol for each biospecimen is described in the supplementary section.

### Digital pathology repository

**Fig. 2:**
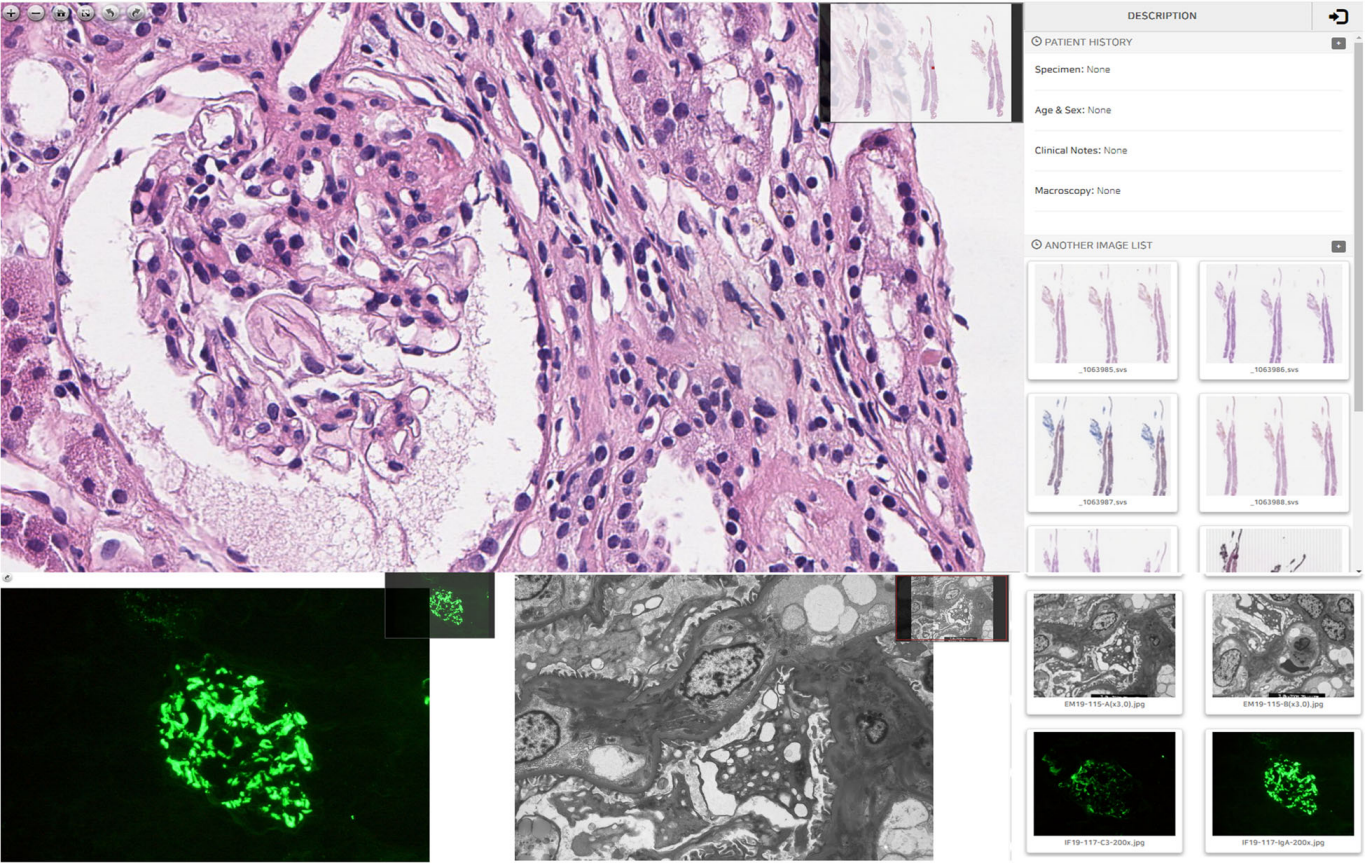
Digital pathology repository. LM, light microscopy; IF, immunofluorescence; EM, electron microscopy

Kidney biopsy materials from enrolled patients, including glass slides scanned into high-resolution whole slide images, both stained and non-stained; digital images of immunofluorescence and electron-micrographs; and pathologic reports from each centre will be collected and uploaded into the KORNERSTONE Digital Pathology Repository (Fig. 2). A digitizing pathology workflow was developed (HuminTec, Suwon, Korea). All of the glass slides will be scanned by Aperio AT2 (Leica Biosystems, Germany, Wetzlar). These digital image files will be stored centrally in the KreoNET server (Seoul National University Hospital, Seoul, Korea). From this system, high-quality pathology images can be acquired in a user-friendly manner and readily shared with clinicians. Before the data are sent to the central server, the study number is allocated to the pathologic images to enable the linking of pathologic data with clinical data and biospecimens. All of these images will be connected in the web-based database.

### Web-based database infrastructure for integration of all information

The overall schemes of the KORNERSTONE are described in Fig. 1. All of the laboratory findings, prescribed medications, and results of pathology will be extracted from the EHR through the clinical data warehouse as Excel or csv files to reduce participants’ burden. The extracted files, including laboratory and anthropometric results from each hospital, are automatically converted into a common case-report form through the macro algorithms. When the converted case-report form is uploaded to the database, different variables for each hospital can be standardized to match internal variables in the web database. The electronic web-based database management system was developed (Binarylab, Seoul, Korea). Additionally, the automatic questionnaire response system was developed for this cohort (ONCE Interactive, Seoul, Korea). Through this system, all questionnaires will be implemented using a tablet to allow patients to respond directly, and all responses are automatically stored in the web-based database.

Researchers or clinical research coordinators who want to upload or download data in the web-based database management system should log in only using a virtual private network (VPN) system to protect personal information. VPN accounts are managed by the principal investigator and clinical research associate and are only accessible by KORNERSTONE authorized staff.

### Governance and dissemination policy of the KORNERSTONE

**Fig. 3:**
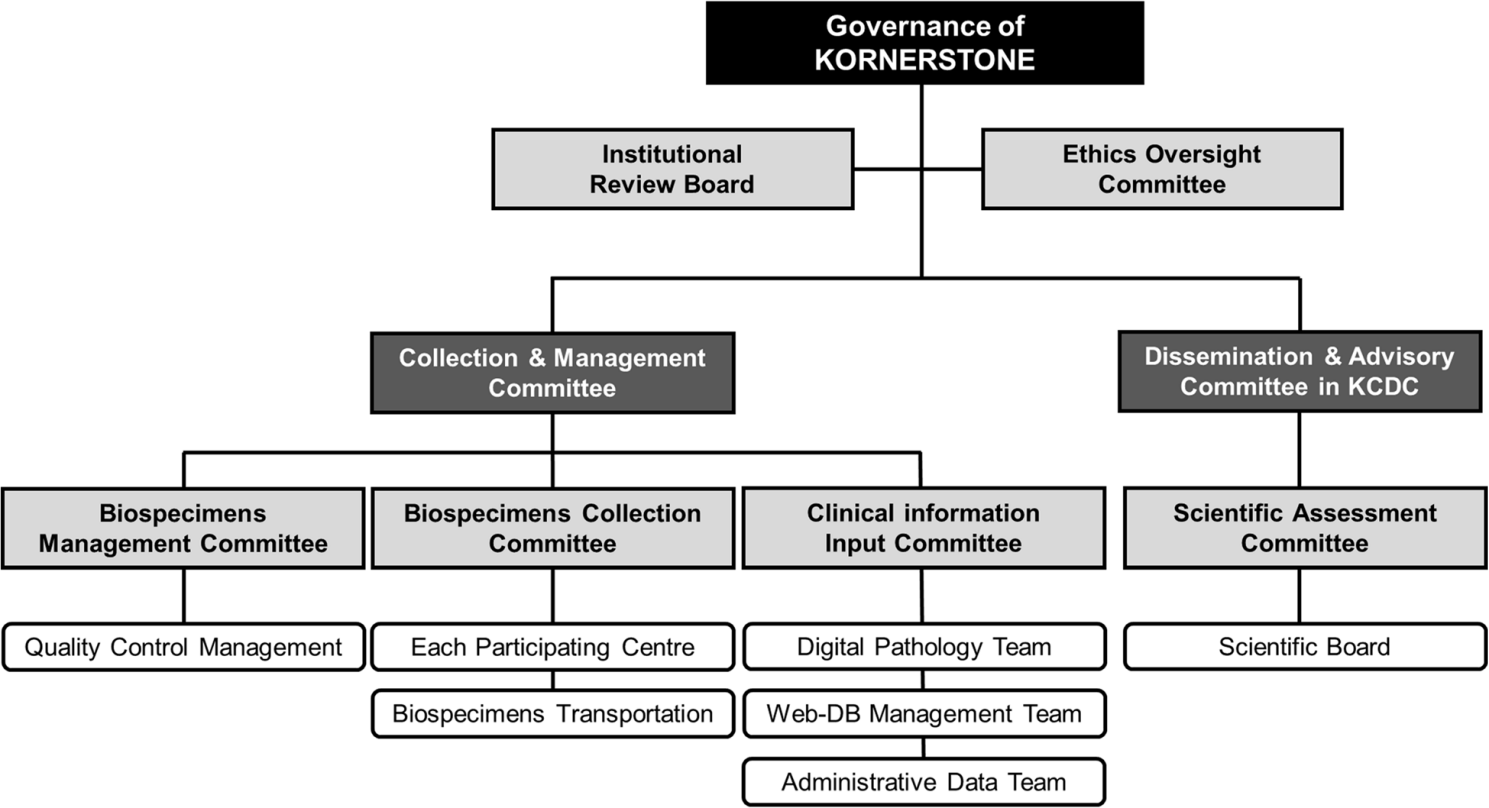
KORNERSTONE governance structure. KORNERSTONE, Korea Glomerular disease Biobank Network; DB, database; KCDC, Korea Centers for Disease Control and Prevention

The purpose of the KORNERSTONE is to provide a foundation for the discovery of new prediction markers of glomerular diseases and the promotion of the utilization of human-derived resources within the legal and ethical regulations. Given the guidelines of future activities, the creation of governance in the KORNERSTONE is crucial. All collection of biospecimens and information and clinical and research activities will be supervised by the independent governance of KORNERSTONE (Fig. 3).

**Fig. 4:**
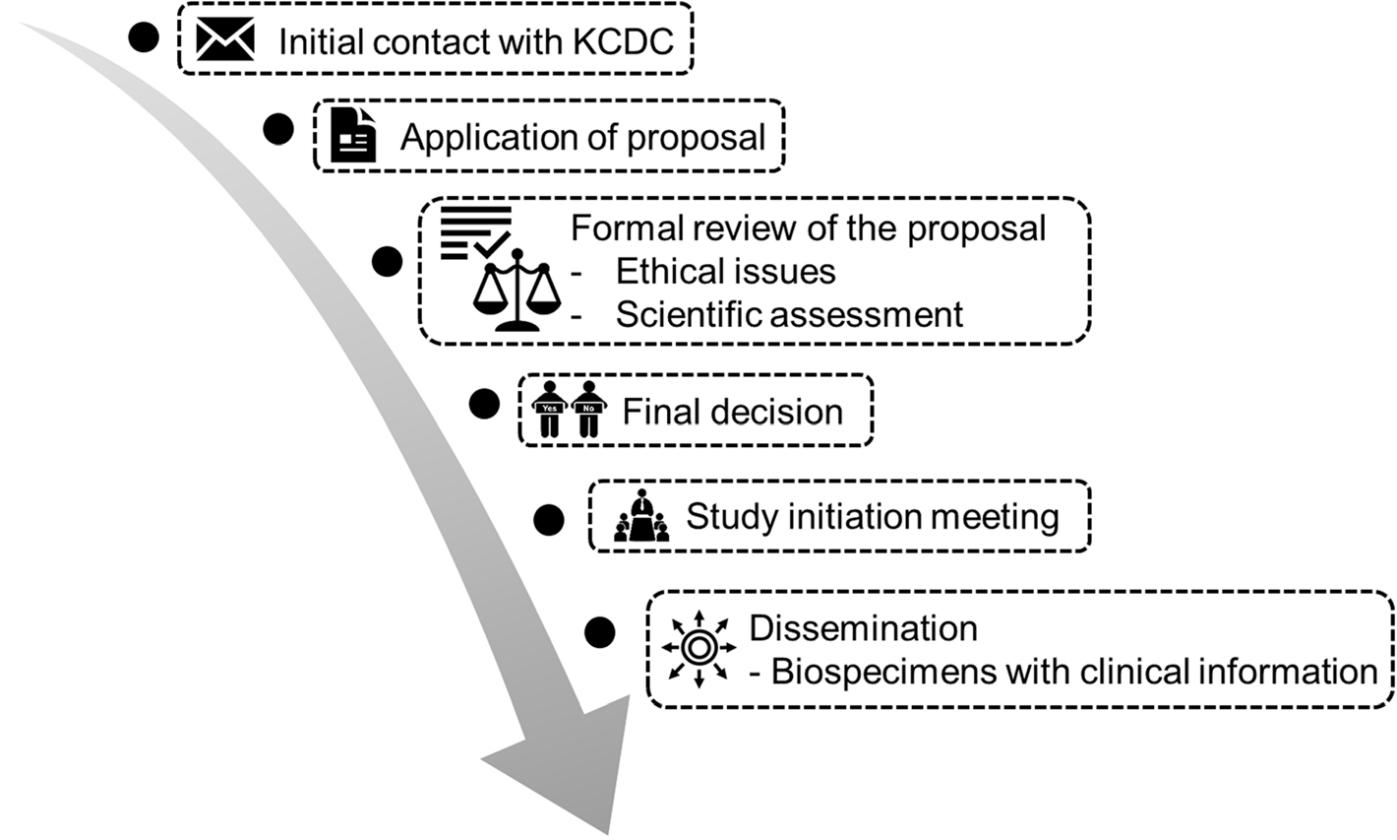
Application and dissemination process of the KORNERSTONE. KORNERSTONE, Korea Glomerular disease Biobank Network; KCDC, Korea Centers for Disease Control and Prevention

Researchers who want to use resources from the KORNERSTONE should contact initially the KCDC (biobank@korea.kr). All of the collected data and biospecimens of the KORNERSTONE can be used only for future studies that are within the perspective of scientific objectives of the KORNERSTONE and that are approved by the Dissemination and Advisory Committee (Fig. 4). Any researchers can submit a study proposal including study objectives and the type of data and biospecimens required to the Dissemination and Advisory Committee of the KORNERSTONE. If a study proposal is approved, a new VPN account will be given to the approved researcher. All of the data will not be accessible, as only information and biospecimens from those patients who meet the inclusion criteria in the approved protocol will be provided to the investigators. The results of each study will be reviewed by the Scientific Assessment Committee of KORNERSTONE and will be published after peer review.

## Discussion

Glomerular diseases, a set of debilitating and complex clinical disease entities affecting individuals of all ages, are associated with significant mortality and morbidity, including hospitalization and ESRD. Although there are many advances in understanding novel genetic and environmental factors affecting the pathophysiology of various glomerular diseases, there is an insufficient number of basic and translational studies that integrate clinical, pathological, and genetic information. The KORNERSTONE, a prospective cohort study with various biospecimens funded by the KCDC, was established for supporting and providing high-quality clinical information and biospecimens based on deep clinical phenotypes of glomerular diseases.

The presentation and prognosis of each glomerular disease covers a wide spectrum even in the same diagnosis, and it is challenging to define each glomerular disease using only clinical descriptions [13]. This fact might be due to the classification of glomerular disease based on histopathology rather than an understanding of the molecular, genetic or environmental underpinnings of these diseases; thus, kidney biopsy is essential for a definitive diagnosis. However, a kidney biopsy is an invasive, burdensome procedure for patients, so multiple biopsy tissue collections are onerous. Misclassification of the subgrouping of glomerular diseases due to the limited reproducibility of pathologic diagnosis is another barrier for studying glomerular disease [14, 15]. While pathological review is important in diagnosis, progression or remission is identified by clinical phenotypes, including proteinuria or eGFR. Considering the chronic pathway of glomerular diseases, a deep clinical phenotype linked serially to obtained biospecimens will be a complementary substitute for the gap between pathology and clinical phenotype regarding time and precision. Thus, in-depth knowledge of each of these glomerular diseases is needed to provide effective care for the patients. Based on this background, many researchers agreed that the practice of personalized and precision medicine is necessary for this disease entity [16]. Precision medicine proposes the development of new disease definitions achieved by a multilayered multi-omics analysis of the disease course [17]. These kinds of studies provide comprehensive information along with the genotype-phenotype continuum, and it is inevitable to establish a systemic biobank that includes well-organized clinical data, evaluation of environmental factors and collection of biospecimens. From this point of view, several prospective observational cohort studies for glomerulonephritis were established: the Toronto Glomerulonephritis Registry, [18, 19] the University of North Carolina Glomerular Disease Collaborative Network, [20, 21] PodoNet, [22, 23], the British Columbia Glomerulonephritis Registry [24], Cure Glomerulonephropathy (CureGN), [25] and the NEPTUNE [5]. In addition, regarding biobanks, the Biobank for the Molecular Classification of Kidney Disease (BMCKD) is underway in Canada [26].

Pathology is a crucial test for the diagnosis and evaluation of prognosis for glomerular diseases. Expertise, abundant experience and reproducibility are critical to exact diagnostic accuracy. A digital pathology repository will be an essential tool for integrating clinical and pathological information together, and this system was introduced in this study. Through this digital pathology, misclassification for a subgroup of glomerular diseases can be reevaluated by many different pathologists and nephrologists. Additionally, with the advancement of high-resolution scanners, computing power, faster networks and file compression technology to scan glass slides, virtual slides with the digitalization of whole pathologic images have been achieved [27]. The introduction of digital pathology allows clinicians and pathologists to efficiently discuss various pathologic findings, not only text-based information. In addition, the reproducibility of diagnosis has been improved. Artificial intelligence that was utilized mainly in radiology and cardiology has been extended to pathology through digital glass slides. Advances in digital pathology and computer-aided diagnostic tools have made it possible for pathologists and nephrologists to identify unique imaging markers related to specific diseases, which leads to improvement of early detection and selection of effective treatment targets [28]. In line with this background, the KORNERSTONE will integrate digital pathologic images.

The KORNERSTONE will collect data from all patients undergoing kidney biopsy due to suspected glomerular disease, which is not limited to a specific diagnosis. Therefore, this cohort will be composed of more diverse diseases, including minimal change disease, focal segmental glomerulosclerosis, membranous nephropathy, membranoproliferative glomerulonephritis, crescentic glomerulonephritis, IgA nephropathy, and lupus nephritis, than the CureGN [25] and NEPTUNE cohorts [5]. Most of the glomerular disease cohorts have been constructed in Western countries. However, the epidemiology of glomerular diseases is different from that in Western populations, [11, 29] so the KORNERSTONE will be a good resource for providing Asian population data and revealing regional variation in glomerular diseases. Additionally, several studies reported that environmental factors and the microbiome can affect the development and prognosis of glomerular diseases, especially in IgA nephropathy [30–32]. The NEPTUNE cohort collected only quality of life and self-reported health data through questionnaires as patient-centred outcomes, and there were no surveys on influential factors other than outcomes. In the KORNERSTONE, environmental factors, including dietary data will be collected using semi-FFQ and food diaries, and stool samples will be gathered for further analysis of the microbiome. This approach will allow many researchers to evaluate in additional detail the impact of environmental factors on glomerular diseases.

One of the strong points of this cohort, administrative data from various government institutions, including HIRA, NHIS, Statistics Korea and the ESRD registry, linked by social security number will be collected. In particular, Korea achieved nationwide health insurance covering its whole population in 1989 [33], and all medical billing data for the total Korean population is included in NHIS and HIRA data. Researchers can achieve full inspection of clinical hard outcomes. Through this method, it is possible to identify all of the clinical outcomes even when the outcomes occur in other hospitals and for patients not enrolled in the clinical centre. Last, after the enrolment, each time the participant visits the hospital, we will check whether they reached the clinical outcomes. Even if there is no event, we collect clinical data and biospecimens every year. Through this follow-up plan, all of the clinical outcomes will be confirmed as soon as possible, and small differences will be detected that are clinically meaningful. Importantly, we can collect diverse and serial biospecimens. In this regard, it is possible to perform further analysis for risk factors of a rapid deterioration of renal function and refine the natural course of each glomerular disease.

In conclusion, we describe the objectives and clinical protocol for the KORNERSTONE. As the first large-scale glomerulonephropathy cohort study with the integration of clinical data, biospecimens and digital pathologic images in Korea, the KORNERSTONE will help to clarify the natural course, complication profiles, and novel treatment targets of the Asian population with glomerular disease. We expect that the KORNERSTONE will promote collaborative research for treatment targets for improving clinical and patient-oriented outcomes in patients with glomerular disease.

## Supplementary information

**Additional file 1.** Human biospecimen collection, quality control and standard operating procedure for each biospecimen

## Data Availability

All of the collected data and biospecimens of the KORNERSTONE can be used only for future studies that are within the perspective of scientific objectives of the KORNERSTONE and that are approved by the Dissemination and Advisory Committee.

## Abbreviations

KORNERSTONE: KOrea Renal biobank NEtwoRk System TOward NExt-generation analysis; complementary DNA, cDNA
CKD: Chronic kidney disease
ESRD: End-stage renal disease
EHR: Electrical health record
KCDC: Korea Center for Disease Control and Prevention
MDHR: Minimum detectable hazard ratio
eGFR: Estimated glomerular filtration rate
HIRA: Health Insurance Review and Assessment Service
NHIS: National Health Insurance Service
ICD-10: International Statistical Classification of Diseases and Related Health Problems-10
IDMS: Isotope dilution mass spectrometry
CKD-EPI: Chronic Kidney Disease Epidemiology Collaboration
KDQOL-SF: Kidney Disease and Quality of Life Short Form
semi-FFQ: Semi-food frequency questionnaires
SOP: Standard operating procedure
VPN: Virtual private network
