# Supplementary material for: Biobanking for glomerular diseases: a study design and protocol for KOrea Renal biobank NEtwoRk System TOward NExt-generation analysis (KORNERSTONE)
Source: BMC Nephrol. 2020 Aug 26;21:367. doi: 10.1186/s12882-020-02016-z (PMC7448429; doi:10.1186/s12882-020-02016-z)
Supplement: Supplementary file 1 — Additional file 1. Human biospecimen collection, quality control and standard operating procedure for each biospecimen [file 12882_2020_2016_MOESM1_ESM.docx]

**Biobanking for glomerular diseases: a study design and protocol for KOrea Renal biobank NEtwoRk System TOward NExt-generation analysis (KORNERSTONE)**

**Corresponding author:**

Dong Ki Kim (dkkim73@gmail.com)

**Contents**

**Human biospecimen collection, quality control and standard operating procedure for each biospecimen** …………………………………………………………………..…….…………2

**Human biospecimen collection, quality control and standard operating procedure (SOP)**

***Genomic DNA***

Genomic DNA will be extracted from blood using a Gentra Puregene kit (Qiagen, GmbH, Hilden, Germany). A total of 6 cc of blood stored in EDTA tubes will be centrifuged at 1,300 ×g at 4°C for 10 minutes. For red blood cell (RBC) lysis, the plasma layer will be removed, and 150-250 μL of buffy coat will be pipetted and transferred to a 15 mL centrifuge tube. To the buffy coat, RBC lysis solution will be added and left for 10 minutes at RT. Then, the samples will be centrifuged at 2,000 ×g for 5 minutes. The rest of the supernatant, except 100-200 μL, will be removed, and the tubes will be vortexed vigorously to resuspend the pellet in the residual fluid. After adding 3 mL of cell lysis solution, the cells will be vortexed vigorously for 20 sec for lysis and centrifuged at 5,000 ×g for 5 minutes. For DNA precipitation, the supernatants will be transferred to a 15 mL tube and gently inverted 50 times after adding 3 mL of isopropanol. After centrifuging at 2,000 ×g for 3 minutes, the supernatants will be discarded. Ten millilitres of 70% ethanol will be added to the tube, and the tube will be inverted several times to wash the DNA pellet. After centrifuging at 2,000 ×g for 1 minute, the supernatant will be removed carefully, and the tube will be drained on a clean piece of absorbent paper, taking care that the pellet remains in the tube. After 15 minutes of air drying, a DNA hydration solution will be added, and the tube will be incubated. The extracted genomic DNA will be stored at -80°C with an anonymous barcode.

For all genomic DNA samples, the OD (optical density) 260/OD280 and OD260/OD230 ratios will be examined. Ten percent of DNA resources will be randomly extracted to check DNA stability and microbial contamination.

***RNA extraction from kidney cortical tissues***

Fresh kidney biopsy cortical tissues will be stored with RNA later and immediately transferred to the laboratory on ice. If micro-dissection is delayed, the tissues will be stored in a deep freezer. Microdissection will be carried out manually under a stereomicroscope using 30 gauge needles for separation of glomeruli and renal tubules.

For glomerular RNA extraction, microdissected tissues will be dissolved in lysis buffer (RLA buffer, 1:50 beta-mercaptoethanol and 4.5 μL of carrier RNA). After pipetting more than 50 times, 70% ethanol will be added and mixed, then transferred to another column and centrifuged at 10,000 rpm for 30 seconds. Samples will be purified further with an RNA Micro Kit (Qiagen GmbH, Hilden, Germany) according to the manufacturer’s instructions.

For tubulointerstitium RNA extraction, microdissected tissues will be dissolved in lysis buffer (RLA buffer and 1:50 beta-mercaptoethanol) and homogenized. After adding 350 μL of RDA and incubating at 70°C for 3 minutes, the suspension will be centrifuged at 18,000 rpm for 10 minutes. All the supernatant except the pellet will be transferred to a new tube and then mixed through pipetting with 200 μL of 95% ethanol. After that, samples will be purified further with an RNA Mini Kit (Promega, Wisconsin, USA).

The purity of RNA will be inspected through the OD260/OD280 ratio of more than 1.6 in 10% of the samples. If the OD260/OD280 ratio is less than 1.6, we will perform an RNA stability test through 28S:18S band ratio and RNA integrity number (RIN) determination.

***Synthesis of cDNA from extracted RNA***

cDNA will be synthesized using a Reverse Transcription System kit (Promega, Wisconsin, USA). Along with 1.2 kb kanamycin positive control RNA, poly(A) and extracted RNA will be added to the microtube and incubated at 70°C for 10 minutes. The sample tube will be centrifuged at 18,000 rpm for 10 minutes and placed on ice. The following reagents will be mixed to a total of 20 μL: 4 μL of 25 mM magnesium chloride, 2 μL of reverse transcription 10X buffer, 2 μL of 10 mM dNTP mixture, 0.5 μL of recombinant RNasin ribonuclease inhibitor, 15 μL of AMV reverse transcriptase and 0.5 μL of oligo(dT)15 primer. Samples will be incubated (42°C, 15 minutes), heated (95°C, 5 minutes) and incubated once more (0-5°C, 5 minutes). After dilution of the first-strand cDNA synthesis reaction with 100 μL of nuclease-free water, PCR amplification will be performed using template-specific upstream and downstream primers. The synthesized cDNA will be stored at -80°C with an anonymous barcode.

***Stool DNA***

We will collect 3 bottles of stool from each participant, containing at least 1 g. Stool DNA will be extracted using a QIAamp DNA stool (Qiagen, GmbH, Hilden, Germany). Stool samples stored at -80°C will be brought to room temperature 30 minutes before extraction, and 200-250 mg of stool will be placed in a 2 mL U-tube on ice. Then, inhibitEX will be added to the U-tube and vortexed continuously for 1 minute before heating the suspension for 5 minutes at 90°C. After that, the samples will be vortexed for 15 seconds and centrifuged at 14,000 rpm for 1.5 minutes to pellet stool particles. Then, 15 μL of proteinase K, 200 μL of supernatant, and 200 μL of AL buffer will be added in order in a newly prepared 2 mL U-tube, vortexed for 15 seconds, and heated at 70°C for 10 minutes. Next, 200 μL of ethanol (96-100%) will be added to the lysate and mixed by vortexing. A new QIAamp spin column will be placed in a 2 mL U tube, and the complete lysate will be carefully applied without moistening the rim. After centrifugation at 14,000 rpm for 1.5 minutes, the tube containing the filtrate will be discarded. The column will be placed in a new 2 mL collection tube, and 500 μL of AW1 buffer will be added and centrifuged at 14,000 rpm for 15 minutes. Another new 2 mL collection tube will be used, 500 μL of AW2 buffer will be added and centrifuged in the same way, and all of the filtrate will be removed. The final extracted DNA will be stored in a deep freezer (-80°C) in an 81-hole cryo paper box with anonymous barcodes.
